# MRI of Nitrous Oxide-Related Subacute Cervical Myelopathy

**DOI:** 10.5334/jbsr.2347

**Published:** 2021-04-08

**Authors:** Leo Vael, Van Walleghem Phyllis, Özkan Özsarlak

**Affiliations:** 1 UZA/AZ Monica, BE; 2 AZ Monica, BE

**Keywords:** myeloneuropathy, nitrous oxide, misuse, toxicity, MRI, N_2_O

## Abstract

**Teaching point:** Myelopathy may occur following nitrous oxide (N_2_O) misuse, even if vitamin B12-levels are normal. The typical appearance on magnetic resonance imaging (MRI), is an inverted V-shaped T2-weighted hypersignal in the dorsal columns of the cervicothoracic spinal cord.

## Case

**Figure 1:**
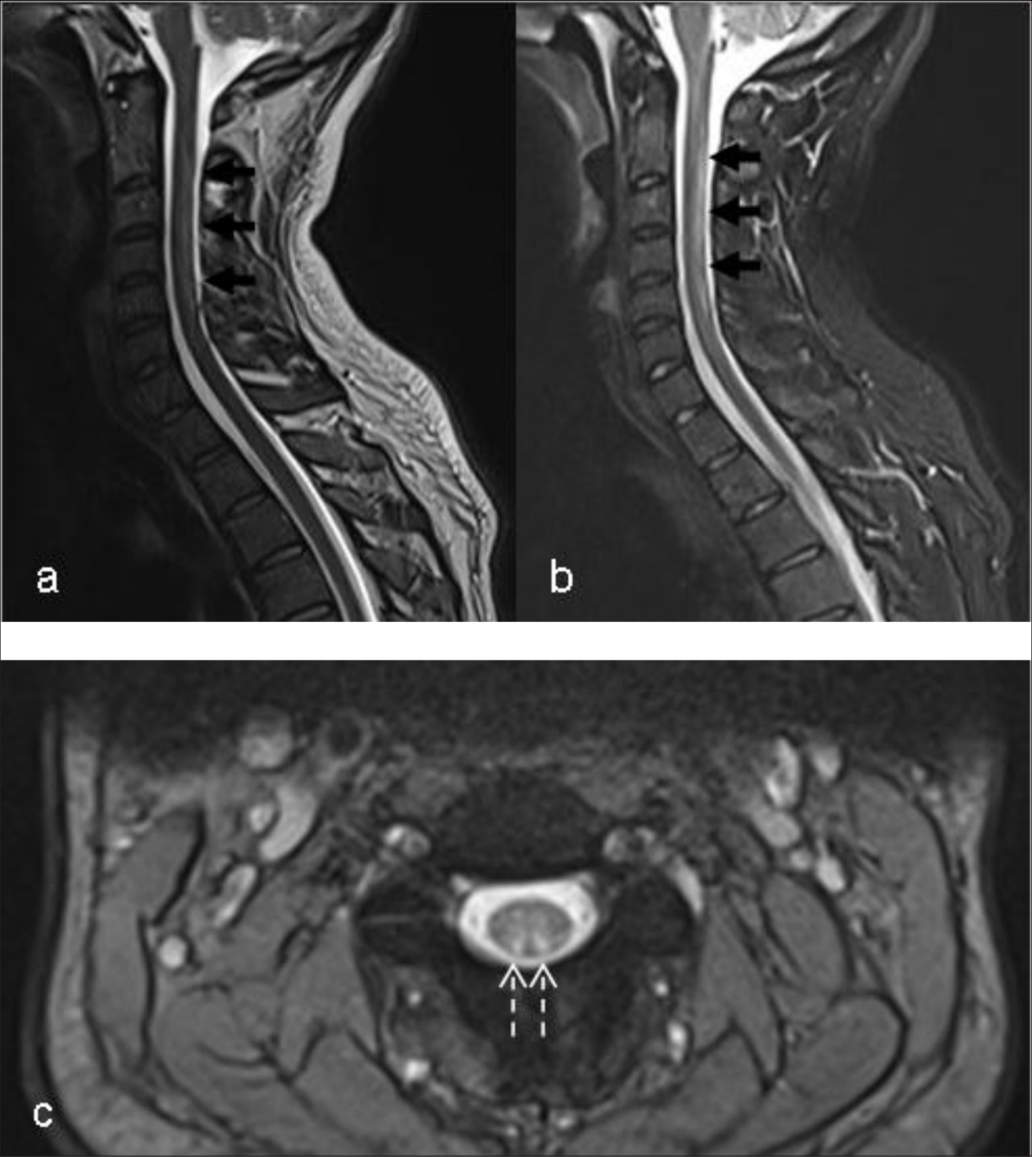


A 30-year-old male was admitted to the emergency department with paresthesia in both upper and lower limbs and gait disturbances. He eventually admitted nitrous oxide (N_2_O, ‘laughing gas’) recreational abuse. Besides this, he also complained of hypersomnia, general weakness, exhaustion, and lack of concentration. Laboratory examination of blood showed normal vitamin B12 levels. Neurological examination revealed paresthesia in upper and lower limbs, slight sensory ataxia, and steppage gait. To confirm the suspected medullary abnormalities, we performed magnetic resonance imaging (MRI) of the spinal cord that showed longitudinal (V-shaped) T2-weighted hyperintensities on both dorsal columns of the cervical spine (***Figure 1***, arrows). Additional brain MRI showed no abnormality. Electromyogram showed a (sub)acute length-dependent axonal motor polyneuropathy with normal sensory neurography. Intramuscular vitamin B12 and folic acid substitution during hospitalization were followed by oral substitution. Nitric oxide use was prohibited. The patient was also admitted to a gait revalidation program. The neurological symptoms rapidly decreased within a few days.

## Discussion

Nitrous oxide is easily approachable in most countries, with a rise of recreational misuse reported in different countries as a “safe” party drug. Nitrous oxide toxicity is nevertheless a known cause of predominantly sensory myeloneuropathy due to vitamin B12 inhibition that causes subacute demyelination and gliosis. Recent reports pointed out to a vitamin-B12 independent toxicity, as the vitamin B12 levels may remain normal as in our case. Imaging therefore plays an important role in diagnosis with inverted V-shaped T2-weighted hyperintense signal involving both dorsal columns of the cervicothoracic spine spinal cord on MRI being typical for N_2_O myelopathy. Hyperintensities in the lateral corticospinal tracts or spinothalamic tracts are less typical features of N_2_O myelopathy. The main differential diagnoses include functional vitamin B12, vitamin E and copper deficiency, which have identical radiographic features and are differentiated by clinical presentation. Other differential diagnoses include demyelinating diseases such as multiple sclerosis, which usually show shorter hyperintense lesions on MRI, and transverse myelitis, which affects lateral and anterior columns of the spine with longer involvement. Detection of subacute nitrous oxide myelopathy within the first few months of symptom onset or in still asymptomatic patients, followed by the initiation of adequate treatment, can invert radiographic features and prevent permanent neurological damage [1].

## References

[1] Keddie S, Adams A, Kelso ARC, et al. No laughing matter: Subacute degeneration of the spinal cord due to nitrous oxide inhalation. J Neurol. 2018; 265(5): 1089–1095. DOI: 10.1007/s00415-018-8801-3 29502317 PMC5937900

